# Association between *ALDH2* polymorphism and esophageal cancer risk in South Koreans: a case-control study

**DOI:** 10.1186/s12885-021-07993-4

**Published:** 2021-03-09

**Authors:** Chang Kyun Choi, Jungho Yang, Sun-Seog Kweon, Sang-Hee Cho, Hye-Yeon Kim, Eun Myung, Min-Ho Shin

**Affiliations:** 1 Department of Preventive Medicine, Chonnam National University Medical School, Hwasun, Republic of Korea; 2 Graduate School of Public Health, Chonnam National University, Gwangju, Republic of Korea; 3 Department of Hemato-Oncology, Chonnam National University Hwasun Hospital, Hwasun, Republic of Korea; 4 Gwangju-Jeonnam Regional Cardiocerebrovascular Center, Chonnam National University Hospital, Gwangju, Republic of Korea; 5 Department of Internal Medicine, Chonnam National University Hospital, Gwangju, Republic of Korea

**Keywords:** *ALDH2*, Alcohol drinking, Case-control studies, Esophageal cancer

## Abstract

**Background:**

Alcohol consumption is a major risk factor for esophageal cancer; however, a high incidence of esophageal cancer is observed particularly among Eastern Asians, although they consume relatively less alcohol, presumably due to the high frequency of aldehyde dehydrogenase 2 (*ALDH2*) rs671 polymorphisms. Nevertheless, the association between *ALDH2* polymorphisms and esophageal cancer remains under debate. In the present study, we evaluated the association between *ALDH2* rs671 polymorphisms and the risk of esophageal cancer in the South Korean population.

**Methods:**

This study included 783 hospital based-cases and 8732 population-based controls. Information on smoking history and alcohol consumption was obtained from the medical records or interview questionnaires. Age-adjusted logistic regression analysis was performed to assess the association between *ALDH2* rs671 polymorphisms and esophageal cancer.

**Results:**

Odds ratios (ORs) for esophageal cancer in men with GA and AA genotypes were 2.75 (95% confidence interval [CI]: 2.34–3.23) and 0.08 (95% CI: 0.00–0.35), respectively; whereas, in women, these ratios were 2.99 (95% CI: 1.43–6.34) and 6.18 (95% CI: 1.40–19.62), respectively, taking subjects with the *ALDH2* GG genotype as a reference. In men, the association between *ALDH2* polymorphisms and esophageal cancer was modified by alcohol consumption.

**Conclusion:**

In Eastern Asians, *ALDH2* rs671 polymorphisms are associated with esophageal cancer, which may be linked to acetaldehyde accumulation.

## Background

Alcohol consumption is a primary risk factor for esophageal cancer. Despite limited alcohol consumption [1], the incidence of esophageal cancer in Eastern Asia is high [2]. Moreover, in contrast to other populations, a characteristic association between less alcohol consumption and high risk of esophageal cancer is observed in Asians [3]. These epidemiological data suggest that Eastern Asians are more susceptible to the carcinogenic effects of alcohol.

The enzyme aldehyde dehydrogenase (ALDH) produces acetaldehyde from alcohol. Acetaldehyde is toxic, and its accumulation is the primary cause of unfavorable symptoms occurring after alcohol consumption. Several enzyme polymorphisms involved in alcohol metabolism affect the degree of acetaldehyde accumulation after alcohol consumption, which varies among individuals of different ethnicities [4]. Aldehyde dehydrogenase 2 (*ALDH2*) rs671 polymorphisms are particularly common among East Asians, and affect alcohol metabolism [5–8]. Compared to individuals with functional ALDH2, inactive ALDH2 carriers present high acetaldehyde levels after alcohol consumption [9], which can lead to flushing, headache, palpitations, and other unfavorable symptoms that affect alcohol consumption habits; thus, minor allele A of *ALDH2* rs671 polymorphisms is associated with low alcohol consumption and alcohol abstinence.

Although the *ALDH2* rs671 GA genotype has been associated with a high risk of esophageal cancer [10–13], this association could not be replicated by certain studies [14, 15]. Furthermore, the relationship between *ALDH2* polymorphisms and esophageal cancer has not been assessed in the South Korean population to date. Therefore, the present study aimed to evaluate the association between *ALDH2* rs671 polymorphisms and the risk of esophageal cancer in South Koreans.

## Methods

### Study population

The case group was consecutively recruited, and comprised 834 patients with histologically confirmed esophageal cancer diagnosed at the Chonnam National University Hwasun Hospital between April 2004 and September 2014. Patients with secondary or recurrent tumors were excluded. Since control subjects were drawn from a community-based cohort study of adults aged 50 years and older, 51 patients under 50 years of age were excluded from the study. Esophageal cancer was classified into different histological types, such as squamous cell carcinoma and adenocarcinoma. The control group included participants from the Dong-gu study, which is a prospective cohort study of risk factors for chronic diseases including stroke, coronary heart disease, cognitive decline, cancer, and fracture [16]. The Dong-gu study comprised 9260 Korean adults aged 50 years and older. Of these, 8805 participants without a cancer history were included. Moreover, participants with missing data were also excluded from the study; hence, our analysis included data from 783 cases and 8732 controls. All patients and control subjects provided informed written consent to participate in this study at the time of peripheral blood collection.

### Genotyping

Genotyping was performed as previously described [17]. In brief, genomic DNA was extracted from peripheral blood using the QIAamp DNA Blood Mini Kit (Qiagen, Valencia, CA, USA) according to the manufacturer’s instructions. *ALDH2* rs671 polymorphism genotyping was performed via high-resolution melting (HRM) analysis using a Rotor-Gene 6000TM (Corbett Research, Sydney, Australia).

### Covariates

Information on smoking and drinking history in the case group was obtained retrospectively from medical records, and in the control group, obtained from interview questionnaires in the baseline survey of the Dong-gu Study. Patients were categorized as current drinkers or nondrinkers, whereas alcohol consumption in the control subjects was evaluated based on the amount and frequency of alcohol consumption, referring to the Dong-gu study. Accordingly, we defined current drinkers as individuals who reportedly consumed one or more drinks per month, and thus, classified the participants as current drinkers or nondrinkers. Smoking status was categorized as smokers (current or ex-smokers) or nonsmokers.

### Statistical analysis

The baseline characteristics of patients and control participants were assessed based on their sex. Moreover, general characteristics between groups were compared using *t*-test for continuous variables and chi-squared test for categorical variables.

The relationship between *ALDH2* rs671 polymorphisms and esophageal cancer was evaluated via age-adjusted logistic regression analysis. Due to the small sample size of participants with AA genotypes, *ALDH2* rs671 polymorphism was classified as GG and GA/AA. Subjects with the *ALDH2* GG genotype were used as a reference. *P*-values lower than 0.05 were considered as statistically significant. All analyses were performed using R software (version 3.6.3, Vienna, Austria).

## Results

Table 1 summarizes the baseline characteristics of the study participants based on their sex. Esophageal cancer patients were younger than the control subjects for both sexes. The frequency of current drinkers was higher in men than in women. Squamous cell carcinoma was the most common histological type of esophageal cancer, followed by adenocarcinoma.

**Table 1:** Baseline characteristics of cases and controls according to sex

	Men			Women		
	Case (*N* = 751)	Control (*N* = 3477)	*P*-value	Case (*N* = 32)	Control (*N* = 5255)	*P*-value
**Age**	66.9 ± 7.9	66.0 ± 8.0	0.007^†^	70.7 ± 9.3	64.5 ± 8.2	< 0.001^†^
**Current drinker**	430 (57.3)	2203 (63.4)	0.002^†^	6 (18.8)	997 (19.0)	1.000^‡^
**Ever-smoker**	556 (74.0)	2576 (74.1)	1.000^†^	3 (9.4)	190 (3.6)	0.110^‡^
**Histologic type**						
**Squamous cell carcinoma**	716 (95.3)			27 (84.4)		
**Adenocarcinoma**	18 (2.4)			3 (9.4)		
**Other**	17 (2.3)			2 (6.2)		

All values are given as N (%) or mean ± standard deviation

^†^*P*-values were calculated by Student’s t-test or chi-square test

^‡^*P*-values were calculated by Fisher’s exact test

**Fig. 1:**
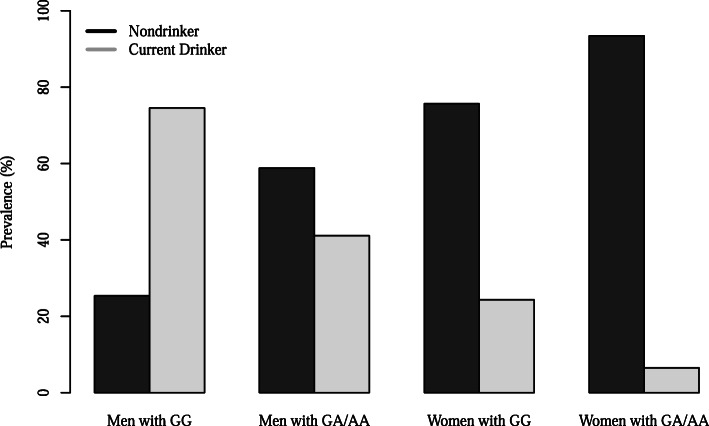
The prevalence of current drinkers according to *ALDH2* rs671 polymorphism and sex

**Fig. 2:**
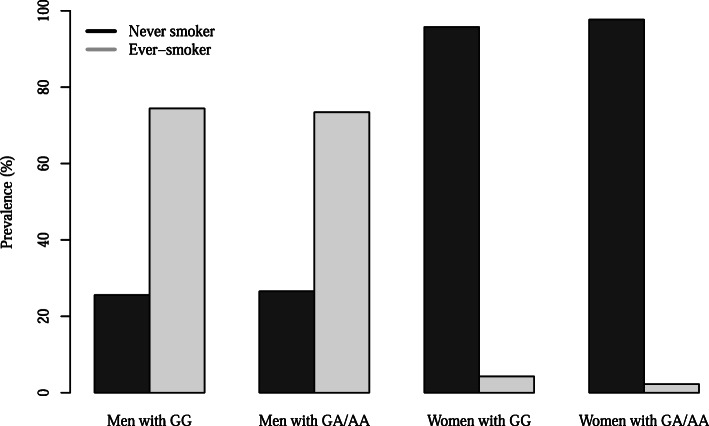
The prevalence of ever-smoker according to *ALDH2* rs671 polymorphism and sex

Figure 1 illustrates the drinking status based on *ALDH2* rs671 genotypes and sex. The prevalence of current drinkers in men with *ALDH2* GG and GA/AA genotypes was 74.6 and 41.1%, respectively; whereas in women, it differed slightly as women with the GG genotype (24.3%) revealed less prevalence than those with the GA/AA genotype (6.5%). In contrast, the prevalence of smoking was not related to the *ALDH2* rs671 genotypes regardless of sex (Fig. 2).

Table 2 summarizes the distribution of *ALDH2* rs671 genotypes, as well as the age-adjusted odds ratio (OR) and 95% confidence interval (CI) for esophageal cancer. Using subjects with the *ALDH2* GG genotype as a reference, ORs for esophageal cancer among male individuals with GA and AA genotypes were 2.75 (95% CI, 2.34–3.23) and 0.08 (95% CI, 0.00–0.35), respectively, whereas those in women were 2.99 (95% CI, 1.43–6.34) and 6.18 (95% CI, 1.40–19.62), respectively.

**Table 2:** Distribution of *ALDH2* rs671 genotype and age-adjusted odds ratio for esophageal cancer according to sex

	Men		Women	
	Case/Control	OR (95% CI)	Case/Control	OR (95% CI)
GG	334/2338	1 (reference)	13/3682	1 (reference)
GA	416/1048	2.75 (2.34–3.23)	16/1440	2.99 (1.43–6.34)
AA	1/91	0.08 (0.00–0.35)	3/133	6.18 (1.40–19.62)
GA + AA	417/1139	2.54 (2.16–2.98)	19/1573	3.25 (1.61–6.76)

*OR* Odds ratio, *CI* Confidence interval

Table 3 summarizes the results of subgroup analysis of the association between *ALDH2* rs671 genotype and esophageal cancer. In current drinkers, *ALDH2* genotypes had a greater impact on esophageal cancer risk than in nondrinkers. In men, the association between *ALDH2* rs671 polymorphisms and esophageal cancer differed significantly based on alcohol consumption (*P* < 0.001). Using subjects with the *ALDH2* GG genotype as a reference, the OR of the *ALDH2* GA/AA genotype among current drinkers was 4.39 (95% CI, 3.54–5.46) in men and 14.45 (95% CI, 2.45–89.48) in women; however, among the nondrinkers, it was 1.25 (95% CI, 0.97–1.61) in men and 2.95 (95% CI, 1.35–6.76) in women. Smoking history did not affect the association between *ALDH2* polymorphisms and esophageal cancer in either sex.

**Table 3:** Distribution of *ALDH2* rs671 genotype and age-adjusted odds ratio for esophageal cancer according to sex, alcohol consumption, and smoking history

	Men		Women	
	Case/Control	OR (95% CI)	Case/Control	OR (95% CI)
Current drinker				
GG	211/1782	1 (reference)	3/896	1 (reference)
GA + AA	219/421	4.39 (3.54–5.46)	3/101	14.45 (2.45–89.48)
Nondrinker				
GG	123/556	1 (reference)	10/2786	1 (reference)
GA + AA	198/718	1.25 (0.97–1.61)	16/1472	2.95 (1.35–6.76)
Ever smoker				
GG	255/1734	1 (reference)	2/155	1 (reference)
GA + AA	301/842	2.41 (2.00–2.91)	1/35	2.02 (0.09–22.02)
Never smoker				
GG	79/604	1 (reference)	11/3527	1 (reference)
GA + AA	116/297	2.95 (2.15–4.07)	18/1538	3.49 (1.67–7.67)

*OR* Odds ratio, *CI* Confidence interval

## Discussion

This is the first study to evaluate the association between *ALDH2* rs671 and esophageal cancer in the South Korean population. Several lines of evidence support the relationship between *ALDH2* rs671 polymorphism and alcohol consumption. In this study, we found that the *ALDH2* GA/AA genotype is associated with a high risk of esophageal cancer. Notably, the *ALDH2* genotype had a higher impact on the risk of esophageal cancer in current drinkers than that in nondrinkers.

In accordance with our findings, previous studies reported a relationship between the *ALDH2* GA/AA genotype and the risk of esophageal cancer. An exploratory genome-wide association study (GWAS) revealed that *ALDH2* rs671 polymorphisms were associated with esophageal cancer [10]. The study also reported that the GA and AA genotypes were associated with high and low risk of esophageal cancer, respectively. Moreover, case-control studies [11, 13] and a prospective cohort study [12] suggested an association between *ALDH2* rs671 polymorphisms and esophageal cancer. In the Kadoorie Biobank cohort [12], individuals with minor allele of *ALDH2* rs671 polymorphism presented high risk of esophageal cancer, and the impact of *ALDH2* polymorphisms on esophageal cancer risk was higher among individuals who consumed alcohol. Nevertheless, these results could not be replicated by other studies, which reported that minor allele of *ALDH2* rs671 polymorphisms were not associated with esophageal cancer [15], or were associated with a low risk of esophageal cancer [14]. These discrepancies could be attributed to differences in average alcohol consumption [1, 5], polymorphism distribution across populations [4], or various alcohol consumption definitions. Furthermore, the relationship between *ALDH2* polymorphisms and alcohol consumption differed based on sex [5], thereby leading to contradictory findings among studies that did not perform sex-stratified analyses. During subgroup analysis for nondrinkers, the *ALDH2* GA/AA genotype revealed a higher risk of esophageal cancer than the *ALDH2* GG genotype in women, unlike in men, presumably due to distinct hormonal receptors in both sexes. ALDH2 may be unable to efficiently detoxify the endogenous aldehydes related to carcinogenesis in the *ALDH2* GA/AA genotype when compared to that in the *ALDH2* GG genotype [18]. It is assumed that the estrogen receptors cause differences in the relationship between *ALDH2* rs671 polymorphism and carcinogenesis [19]; however, the biological mechanism between *ALDH2* and hormonal receptors needs to be further evaluated.

Moreover, differences in exposure to salivary acetaldehyde among individuals with distinct genotypes may impact the effect of the *ALDH2* genotype on esophageal cancer risk. The salivary acetaldehyde levels after ethanol consumption were higher in ALDH2-deficient individuals compared to individuals with functional ALDH2; this difference was observed after consuming two to three drinks [9, 20]. Thus, alcohol consumption can increase the acetaldehyde levels in esophagus, particularly in individuals with *ALDH2* GA/AA genotypes with slow acetaldehyde metabolism. Acetaldehyde associated with alcoholic beverages has been classified as a group 1 carcinogen by the International Agency for Research on Cancer [21]. Acetaldehyde reacts with DNA, generating unstable DNA adducts and inducing DNA damage [22]. In a study involving Japanese alcoholic patients, the *ALDH2* GA genotype was associated with high levels of N2-ethylidene-di-deoxyguanosine, the most common DNA adduct induced by acetaldehyde [23].

The present study has several limitations. First, the number of patients with AA genotypes was small; therefore, we could not perform subgroup analysis to evaluate the risk of esophageal cancer according to the genotype. Second, we did not assess the role of alcohol dehydrogenase 1B (*ADH1B*) rs1229984 polymorphisms, which are common in East Asians. Third, the dose-response relationship between alcohol consumption and esophageal cancer risk could not be assessed, because the amount of alcohol consumption was not evaluated in the case group. It is necessary to assess the relationship between genetically predicted alcohol consumption and esophageal cancer through a two-stage or two-sample Mendelian randomization study.

## Conclusions

*ALDH2* rs671 polymorphisms are associated with esophageal cancer in Eastern Asians. Notably, individuals with *ALDH2* GA/AA genotypes who consumed alcohol were at a particularly high risk of esophageal cancer, presumably due acetaldehyde accumulation in the esophagus. By answering a questionnaire on alcohol flushing response, high-risk individuals with low-activity ALDH2 can be identified noninvasively without genotyping resources [8, 12], and esophageal cancer prevention programs such as interventions for alcohol consumption cessation or screening programs can be effectively implemented [24].

## Data Availability

The datasets used and analyzed during the current study are available from the corresponding author on reasonable request.

## Abbreviations

ALDH: Aldehyde dehydrogenase
OR: Odds ratio
CI: Confidence interval
GWAS: Genome-wide association study
ADH1B: Alcohol dehydrogenase 1B
